# Genomic diversity and *BCL9L* mutational status in circulating tumor cells predict overall survival in metastatic colorectal cancer

**DOI:** 10.1007/s13402-025-01109-x

**Published:** 2025-10-06

**Authors:** Joao M. Alves, Nuria Estévez-Gómez, Roberto Piñeiro, Laura Muinelo-Romay, Patricia Mondelo-Macía, Mercedes Salgado, Agueda Iglesias-Gómez, Laura Codesido-Prada, Astrid Diez-Martín, Joaquin Cubiella, David Posada

**Affiliations:** 1 CINBIO, Universidade de Vigo, Vigo, 36310 Spain; 2 Galicia Sur Health Research Institute (IIS Galicia Sur), SERGAS-UVIGO, Vigo, Spain; 3 Translational Medical Oncology Group, Oncomet, Health Research Institute of Santiago de Compostela (IDIS), Santiago de Compostela, Spain; 4 Centro de Investigación Biomédica en Red de Cáncer (CIBERONC), Madrid, Spain; 5 Liquid Biopsy Analysis Unit, Translational Medical Oncology Group, Health Research Institute of Santiago de Compostela (IDIS), Santiago de Compostela, Spain; 6 Department of Oncology, Hospital Universitario de Ourense, Research Group in Gastrointestinal Oncology-Ourense, Ourense, Spain; 7 Department of Gastroenterology Hospital Universitario de Ourense, Research Group in Gastrointestinal Oncology-Ourense, Centro de Investigación Biomédica en Red de Enfermedades Hepáticas y Digestivas (CIBERehd), Ourense, Spain

**Keywords:** Circulating tumor cells, Liquid biopsy, Intratumor genomic heterogeneity, Colorectal cancer, Precision medicine

## Abstract

**Background:**

Metastatic colorectal cancer (mCRC) remains a major cause of cancer-related mortality, but few noninvasive biomarkers exist to track disease progression or inform treatment strategies. Circulating tumor cells (CTCs) offer a minimally invasive source of tumor material, yet the prognostic significance of their genomic diversity remains unclear.

**Methods:**

We conducted whole-exome sequencing of CTC pools from 29 mCRC patients to characterize their mutational landscape and assess associations with overall survival.

**Results:**

Our analysis revealed substantial variation in mutational burden among patients, with all CTC pools harboring non-silent mutations in key CRC driver genes. Higher genomic diversity in CTC pools was significantly associated with reduced overall survival. Additionally, non-silent mutations in *BCL9L* emerged as a strong predictor of patient survival.

**Conclusion:**

Genomic diversity and *BCL9L* mutational status in CTC pools emerged as strong predictors of survival in mCRC, underscoring the potential of CTC genomic profiling as a minimally invasive and clinically relevant prognostic tool in mCRC.

**Supplementary Information:**

The online version contains supplementary material available at 10.1007/s13402-025-01109-x.

## Introduction

Metastatic colorectal cancer (mCRC) remains one of the leading causes of cancer-related deaths worldwide [1]. Despite encouraging improvements in the detection and treatment of early-stage lesions [2, 3], mCRC continues to have a dismal 5-year survival rate of roughly 14% [4], highlighting the urgent need for biomarkers that can effectively monitor disease progression, guide treatment decisions, and improve patient outcomes [5, 6].

The clinical management of mCRC has improved substantially with the integration of surgery (including hepatectomy and liver transplantation), systemic chemotherapy, and targeted therapies, such as anti-angiogenic agents and anti-EGFR monoclonal antibodies, in molecularly selected patients [7]. Nonetheless, early detection of metastases, treatment monitoring, and timely identification of progression still rely on invasive or costly procedures such as serial imaging and tissue biopsies. These limitations reinforce the need for more accessible tools to support the clinical management and prognostic stratification of mCRC patients [8].

Multiple studies have now demonstrated that tumors with higher levels of genomic diversity are associated with more aggressive disease [9], shorter recurrence times [10], and poorer survival outcomes [11]. However, measuring genomic diversity from solid tissues often involves invasive and high-risk biopsy procedures. As an alternative, liquid biopsies from peripheral blood and other body fluids, such as saliva and urine, offer a minimally invasive procedure for measuring tumoral genomic diversity through the analysis of circulating tumor DNA (ctDNA) and circulating tumor cells (CTCs) [12]. Although most efforts in this regard have focused on the genomic characterization of ctDNA due to its relative ease of isolation [13], the analysis of CTCs provides a unique opportunity to study intact tumor cells, thereby allowing for comprehensive whole-genome profiling of tumors [14]. Moreover, CTCs are presumably responsible for metastatic seeding and can provide information for both primary and metastatic tumor development [15, 16].

In this context, CTC enumeration has already been validated as a reliable prognostic marker across several cancer types [17, 18], including CRC [19, 20]. However, not all CTCs within a patient are genetically identical, and initial sequencing studies have revealed that CTCs can exhibit significant genomic variation within patients [15, 21, 22]. The genomic diversity of a patient’s CTC pool may hold clinical significance for several reasons. First, the CTC pool could serve as a proxy for intratumoral genomic diversity, which, as mentioned above, has already been associated with increased cancer aggressiveness. Second, CTCs are considered precursors of metastasis. As such, a genomically diverse CTC population may increase the potential for successful dissemination, colonization, and growth in new environments [23]. Nevertheless, the clinical significance of CTC genomic diversity remains largely unexplored.

In this study, we evaluated the clinical relevance of CTC genomic variation in mCRC. Using whole-exome sequencing data from CTC pools collected from 29 patients with mCRC, we provide a comprehensive description of the mutational landscape of CTCs and demonstrate that the overall genomic variation of CTC pools and the mutational status of the *BCL9L* gene can help predict patient survival.

## Material & methods

### Clinical cohort and blood collection

We enrolled 25 sporadic mCRC patients diagnosed between October 2017 and January 2022 at the Hospital Universitario de Ourense, Spain, with histologically proven CRC. We collected a 15 mL blood sample for each patient, stored in Transfix CTC-TVT tubes (Cytomark, UK) at room temperature. For patients undergoing chemotherapy treatment, blood samples were obtained immediately before the start of a new chemotherapy cycle.

### CTC enrichment and PBMC isolation

We processed all samples within 96 hours of collection using the Parsortix® platform (ANGLE plc, UK), which traps CTCs due to their larger size and lower compressibility compared to blood cells. For each patient, 10 mL of whole peripheral blood was loaded into a Parsortix microfluidic device. We enriched each sample in disposable Parsortix cassettes with a gap size of 6.5 µm (GEN3D6.5, Angle Inc., Guildford, UK) and at 99 mbar, according to the manufacturer’s guidelines. After separation, the captured cells were collected in 200 µL of PBS and stored at − 80 °C.

To obtain the peripheral blood mononuclear cell (PBMC) fraction -to be used as “healthy controls”- we took the remaining 5 mL from each blood sample and performed Ficoll-Paque gradient centrifugation. We kept the PBMCs in RNAlater (Ambion, TX, USA) at −80 °C until genomic DNA (gDNA) extraction.

### Whole-genome amplification of CTC pools

Given the large collection volume (~200 µl), we initially performed gDNA extraction of the CTC pools using the QIAamp DNA Blood Mini Kit (Qiagen, Germany) before proceeding with whole-genome amplification (WGA) using the Ampli1^TM^ kit (Menarini Silicon Biosystems, Italy). We carried out the WGA starting with 1 µl of gDNA and included positive (10 ng/µl REPLIg human control kit, Qiagen, Germany) and negative controls (DNase/RNase-free water). We worked in a laminar-flow hood to avoid contamination and used a dedicated set of pipettes and UV-irradiated plastic materials. We evaluated the quality of the amplified product using the Ampli1 QC Kit (Menarini Silicon Biosystems, Italy), a PCR-based assay to establish DNA integrity. Samples that produced at least two of the four expected PCR fragments were selected for the following steps: increasing the total double-stranded DNA (dsDNA) with the Ampli1 ReAmp/ds kit (Menarini Silicon Biosystems, Italy) and WGA adaptor removal. The latter was carried out by incubating a mixture of 5 µl of NEBuffer 4 10X (New England Biolabs, MA, USA), 1 µl of MseI 50 U/µl (New England Biolabs, MA, USA), 19 µl of nuclease-free water and 25 µl of dsDNA at 37 °C for 3 h, with a final step of enzyme inactivation at 65 °C for 20 min. Finally, we purified the samples with 1.8X AMPure XP beads (Agencourt, Beckman Coulter, CA, USA), quantified the DNA yield with Qubit 3.0 fluorometer (Thermo Fisher Scientific, MA, USA), and checked the amplicon size distribution with the D1000 ScreenTape System in a 2200 TapeStation platform (Agilent Technologies, CA, USA).

### PBMCs gDNA isolation

In parallel, we used the QIAamp DNA Blood Mini Kit (Qiagen, Germany) to extract gDNA from the PBMCs. We estimated DNA yield using the Qubit 3.0 fluorometer (Thermo Fisher Scientific, MA, USA) and DNA integrity with the Genomic DNA ScreenTape Assay (Agilent Technologies, CA, USA).

### Whole-exome sequencing

Sequencing libraries were constructed at the Spanish National Center for Genomic Analysis (CNAG; http://www.cnag.crg.eu) with the SureSelect XT and Agilent Human Exon v5 kits (Agilent Technologies, CA, USA). In total, 25 amplified CTC pools and 25 PBMCs gDNA samples were whole-exome sequenced (WES) at 100X and 60X, respectively, on an Illumina NovaSeq 6000 (PE100) at CNAG. In addition, we also included matched CTC pools and PBMCs WES datasets previously generated in our lab from four sporadic mCRC patients (“PP”) [24] (Sequence Read Archive accession code PRJNA886718).

### Data processing

After trimming amplification and sequencing adapters from the raw FASTQ files, we aligned the sequencing reads to the Genome Reference Consortium Human Build 37 (GRCh37) using the MEM algorithm in the BWA software [25]. Following the GATKs standardized best-practices pipeline [26], we filtered out reads with low mapping quality, performed a local realignment around indels, and removed PCR duplicates.

### Somatic variant calling

We identified somatic single-nucleotide variants (SNVs) and short insertions and deletions (indels) for each patient using the paired-sample variant-calling approach implemented in MuTect2 software [27] (i.e., CTC pools (tumor) + PBMCs (healthy control)). We then applied GATK FilterMutectCalls to remove calls in problematic sequence contexts (“–orientation-bias-artifact-priors”) or due to potential cross-sample contamination (“–contamination”). Genotypes supported by < 10 total reads, with < 2 alternative reads, or with a variant allele frequency (VAF) ≤ 0.075 were set as missing, and variants composed only of missing genotypes were removed. We annotated the variants with Annovar (v.20200608) [28]. Additionally, for each patient, we estimated somatic copy number gains, losses, loss-of-heterozygosity (LOH) events, tumor purity, and global ploidy status using Sequenza [29] under default settings.

### MSI status

Furthermore, we identified the microsatellite stability status of each patient with MSIsensor-pro [30], following the recommended ‘best practices’ for paired-sampling analysis. We classified patients with more than 30% of microsatellites mutated as microsatellite instable (MSI), while all other patients were categorized as microsatellite stable (MSS), as in Heide et al. [31].

### Mutational signatures

We ran the SIGNAL web tool (https://signal.mutationalsignatures.com/) [32] under default parameters to identify the single base substitution (SBS) signatures active in each patient [33]. As recommended by the authors, SBS fitting was performed using candidate SBS signatures from CRC.

### Estimation of genomic diversity in CTC pools

The genomic diversity of a CTC pool can be measured in multiple ways depending on the type and features of the variants selected. Here, we computed 15 diversity metrics, including mutational burden (MB), the proportion of aberrant genome (PAG) [34], the mutant allele tumor heterogeneity score (MATH) [35], number of SNVs and indels at CRC driver genes, number of copy number alterations (CNAs) at CRC driver genes, and mutation status at recurrently mutated CRC driver genes. Since our cohort included only one MSI patient, all downstream analyses were restricted to the MSS cases (*N* = 28).

#### Mutational burden (MB)

For each patient, we measured the CTC pool mutational burden (MB) as the total number of SNVs that passed our filtering thresholds, normalized by the size of the exome captured. We report MB in units of the number of SNVs/megabase.

#### Proportion of aberrant genome (PAG)

We also measured, for each patient, the proportion of the autosomal genome affected by copy number gains, losses, and LOH events.

#### Mutant allele tumor heterogeneity (MATH)

We estimated for each patient the CTC-pool mutant-allele tumor heterogeneity (MATH) score [35], which is based on the distribution of SNV and indel allelic frequencies and calculated as:

$$MATH = 100 \times {{median\, absolute\, deviation\, \left( {VAF} \right) } \over {median \left( {VAF} \right)}}$$

Where VAF is the variant allele frequency of SNVs and indels. Since MATH scores are sensitive to unreliable VAF at sites with insufficient sequencing depth [36], we only considered SNVs and indels with ≥ 25 total reads and ≥ 5 alternative reads.

#### Number of SNVs and indels at CRC driver genes (CRC-mut)

We additionally measured the total number of non-silent SNVs and indels (i.e., mutations that alter the amino acid sequence of a protein and may affect its function) overlapping with CRC driver genes at the IntOGen portal (release 2023) [37].

#### Number of CNAs at CRC driver genes (CRC-CNA)

We estimated the number of copy number alterations (CNAs), which include gains, losses, and LOH events in the CTC pools overlapping with CRC driver genes at IntOGen (release 2023).

#### Mutation status at CRC driver genes

For CRC driver genes with recurrent non-silent SNVs/Indels in more than four patients, we additionally considered their mutation status (i.e., unmutated vs. mutated) as a diversity metric.

### Correlation of CTC genomic diversity with overall survival

We correlated the above diversity metrics with overall survival (OS). To select which metrics should be included in a multivariate Cox proportional hazards (CPH) analysis, we assessed them individually in a univariate CPH analysis. We used the raw values for the continuous metrics. For the discrete ones (i.e., mutation status and gender), we used “unmutated” and “female” as reference groups, respectively.

Additionally, we created a binary version of each continuous metric (i.e., bMB, bAge, bLoT, bPAG, bMATH, bCRC-mut, and bCRC-CNA) for inclusion in a univariate analysis by splitting the patients into two groups given an optimal threshold. This threshold was determined using CutoffFinder [38], which fits a CPH model to the explanatory (diversity metric) and response (OS) variables, identifying the optimal cutoff as the value that provides the most statistically significant split based on a log-rank test (Table S1).

Adopting a similar strategy to that of Fernandez-Mateos et al. [10], we only included diversity metrics with a p-value ≤0.1 in the multivariate analysis. Importantly, when the continuous and binary versions of a metric returned p-values ≤0.1, only the continuous version was included in the multivariate analysis. All CPH analyses were performed using the survival [39] and survminer [40] R packages.

### Evaluating custom and commercial cancer gene panels for CTC genomic diversity estimation

To identify a cost-effective strategy for the genomic characterization of CTC pools, we constructed and evaluated two customized gene panels, each consisting of a subset of the most frequently mutated genes in our cohort. These panels were tested to determine their ability to provide diversity estimates comparable to those derived from WES. The first panel, CRC-P22, comprised 22 genes that harbored more than 15 mutations across patients. The second panel, CRC-P55, included 55 genes with > 10 mutations across patients. In addition to these custom panels, we evaluated the performance of two commercially available NGS panels, the Pillar® oncoReveal Solid Tumor v2 Panel (48 genes; Pillar Biosciences, MA, USA) and the Oncomine Precision Assay (50 genes; Thermo Fisher Scientific, MA, USA). A list of the genes from the four panels is provided in Table S2.

We extracted the SNVs and indels for each panel and calculated the MATH scores for each patient as in Sect. 2.10.2. Importantly, given the much smaller number of variants in these panels, we did not apply any filter based on read counts, as previously done. Finally, we assessed the clinical relevance of these panels by correlating their derived MATH scores with OS using a univariate CPH analysis. As before, we additionally tested a binary MATH (bMATH) version by dichotomizing patients at optimal threshold values determined using CutoffFinder.

### Declaration of AI-assisted copy editing

During the preparation of this work, we utilized ChatGPT 4.0 Turbo to enhance language and readability. After using this tool/service, we reviewed and edited the content as needed and took full responsibility for the content of the publication.

## Results

### Genomic characterization of CTC pools from the mCRC cohort

**Figure 1:**
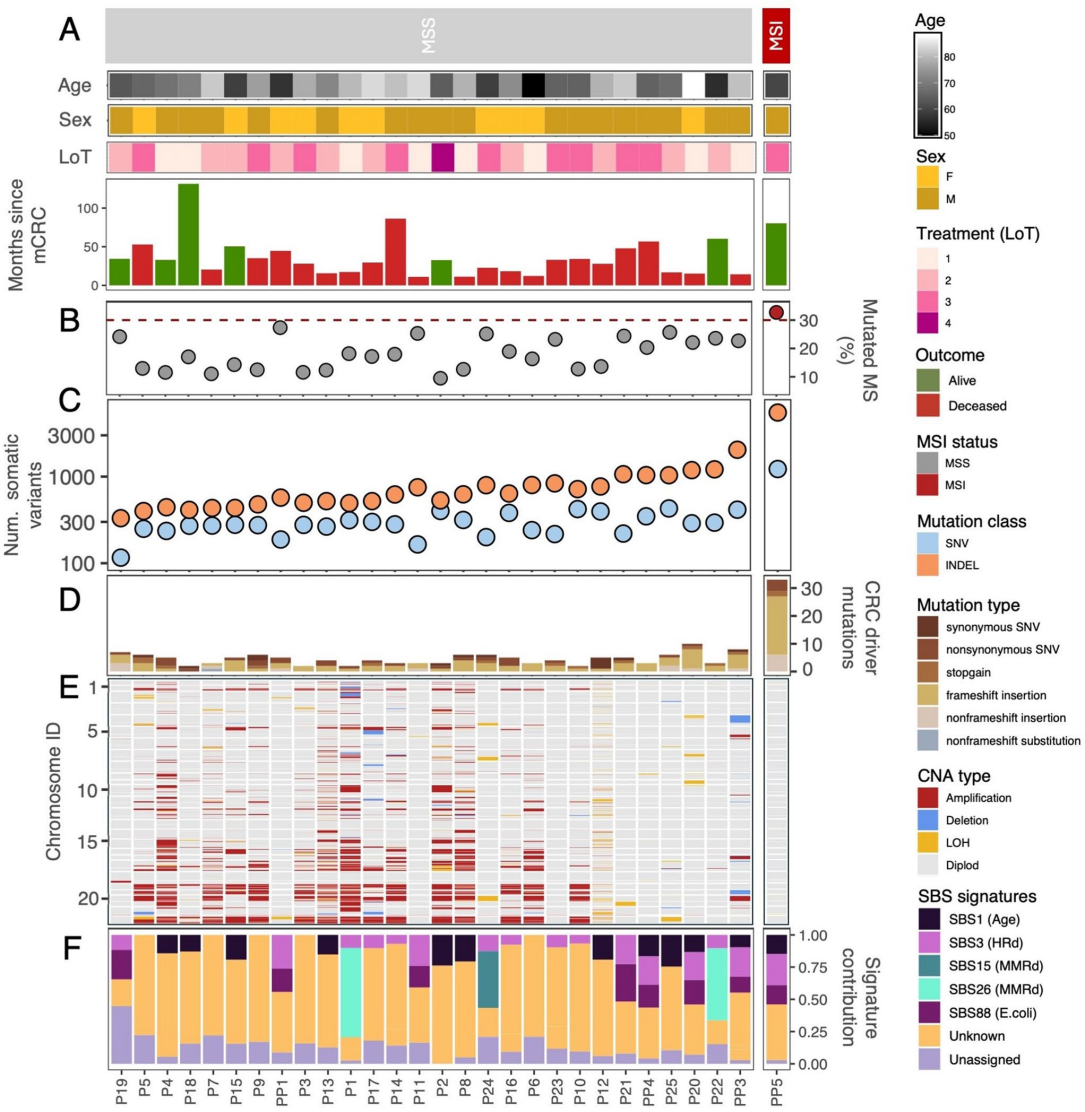
Clinical and genomic characterization of the mCRC cohort. mCRC patients are split based on MS status and ordered according to their total number of somatic variants. **A**. Age, sex, number of lines of treatment (LoT), and months since mCRC diagnosis. **B**. Proportion of mutated microsatellites. Data points are colored by the MS status (grey = “MSS”, red =”MSI”). The dashed red line depicts the threshold for MSI classification (i.e., 30% mutated microsatellites). **C**. Total number of somatic mutations identified with MuTect2, including SNVs (light blue) and indels (orange). **D**. Total number of mutations affecting known CRC driver genes in each patient. Bars are colored according to the type of mutation. **E**. Genome-wide copy number profiles for each patient. Only autosomes are shown, with chromosomes ordered from 1 to 22. Genomic regions are colored according to the type of CNA. **F**. Contribution of the different mutational signatures. HRd = homologous recombination deficiency; MMRd = mismatch repair deficiency. SBS signatures with unknown etiology - SBS8, SBS93, SBS97, SBS121, and SBS123 - were collapsed into a single “Unknown” category

A brief overview of the cohort is presented in Fig. 1A. Comprehensive clinical data for this cohort, including tumor and treatment details, are provided in Table S3. Ten (34.5%) patients were female, and 19 (65.5%) were male, with a mean age at the time of mCRC diagnosis of 66.28 ± 16.33 years.

After sequencing, we identified one patient (PP5) exhibiting microsatellite instability (MSI) (Fig. 1B). As expected, this patient displayed a substantially higher SNV and indel burden (Fig. 1C). Specifically, for PP5, we called a total of 1,224 SNVs and 5,509 indels. In contrast, we estimated 116–429 SNVs and 331–2,043 indels for the other patients.

We identified a large number of mutations in known CRC driver genes, including non-synonymous changes and stop codon gains in *APC*, *KRAS,* and *PIK3CA*. Notably, the number of mutations in CRC driver genes varied significantly within the cohort, ranging from two mutations in patients P1, P3, P10, and P18 (MSS) to 33 mutations in patient PP5 (MSI) (Fig. 1D). We also inferred a substantial number of CNAs, with considerable differences among patients (Fig. 1E). Consistent with previous reports [31], the MSI patient PP5 exhibited a predominantly diploid copy number profile.

We found ten single-base substitution signatures, including signatures commonly observed in CRC, such as SBS1 (age-related), SBS3 (homologous recombination deficiency) and SBS88 (associated with exposure to *E.coli* bacteria) (Fig. 1F). Nevertheless, there was a significant contribution of signatures of unknown etiology - SBS8, SBS93, SBS97, SBS121 and SBS123 - all of which are relatively infrequent in CRC.

### CTC pools show high levels of genomic variation

**Figure 2:**
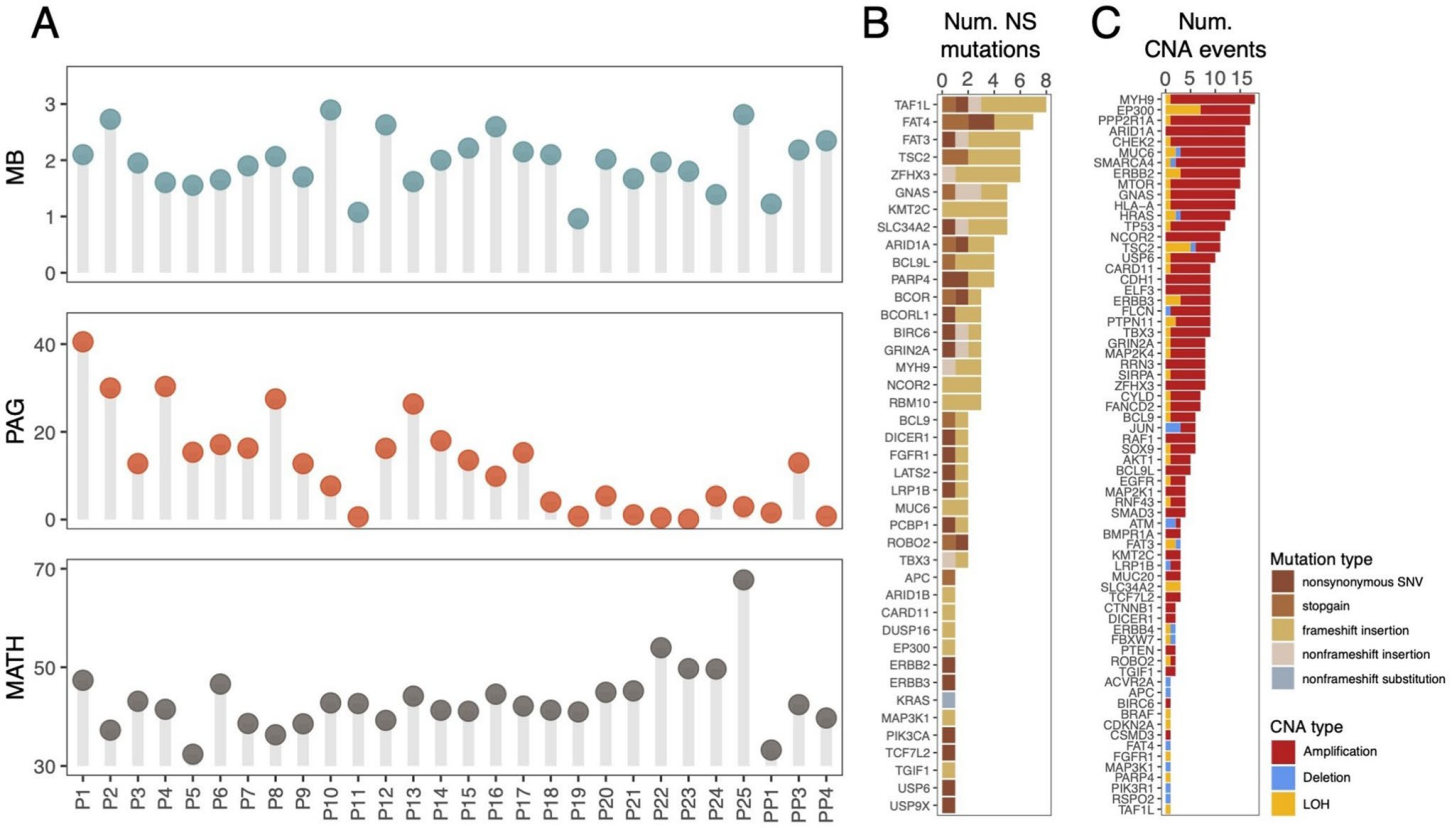
Genomic diversity in CTC pools. **A**. Lollypop plots depict the mutational burden (MB), the proportion of aberrant genome (PAG), and the mutant allele tumor heterogeneity (MATH) score for each patient. **B**. Number of non-silent (NS) SNVs and indels affecting known CRC driver genes. Bars are colored according to the type of mutation. **C**. Number of CNAs overlapping known CRC driver genes. Bars are colored according to CNA type

We observed high levels of genomic variation in the CTC pools (Fig. 2A), with substantial variation among patients. The MB estimates ranged from 0.95 to 2.89 SNVs/Mb, PAG values from 0.05% to 40.5%, and MATH scores between 32.38 and 67.63.

In addition to the substantial variability in the number of non-silent SNVs in CRC driver genes observed among patients (Fig. 1D), we also identified several genes that were recurrently mutated across multiple patients (Fig. 2B). In particular, ten driver genes were independently mutated in at least four patients, including *ARID1A* (N = 4), *BCL9L* (N = 4), *BIRC6* (N = 5), *FAT3* (N = 5), *FAT4* (N = 5), *KMT2C* (N = 5), *PARP4* (N = 4), *TAF1L* (N = 7), *TSC2* (N = 4), and *ZFHX3* (N = 5). Similarly, CNAs encompassing CRC drivers were also widespread (Fig. 2C), with *ARID1A* (N = 16), *MYH9* (N = 17), and *PPP2R1A* (N = 16) exhibiting the highest frequency of copy gains. In contrast, *EP300* (N = 7) and *TSC2* (N = 6) showed the highest number of deletions and LOH events.

### MATH score and BCL9L status predict survival outcome in mCRC

**Figure 3:**
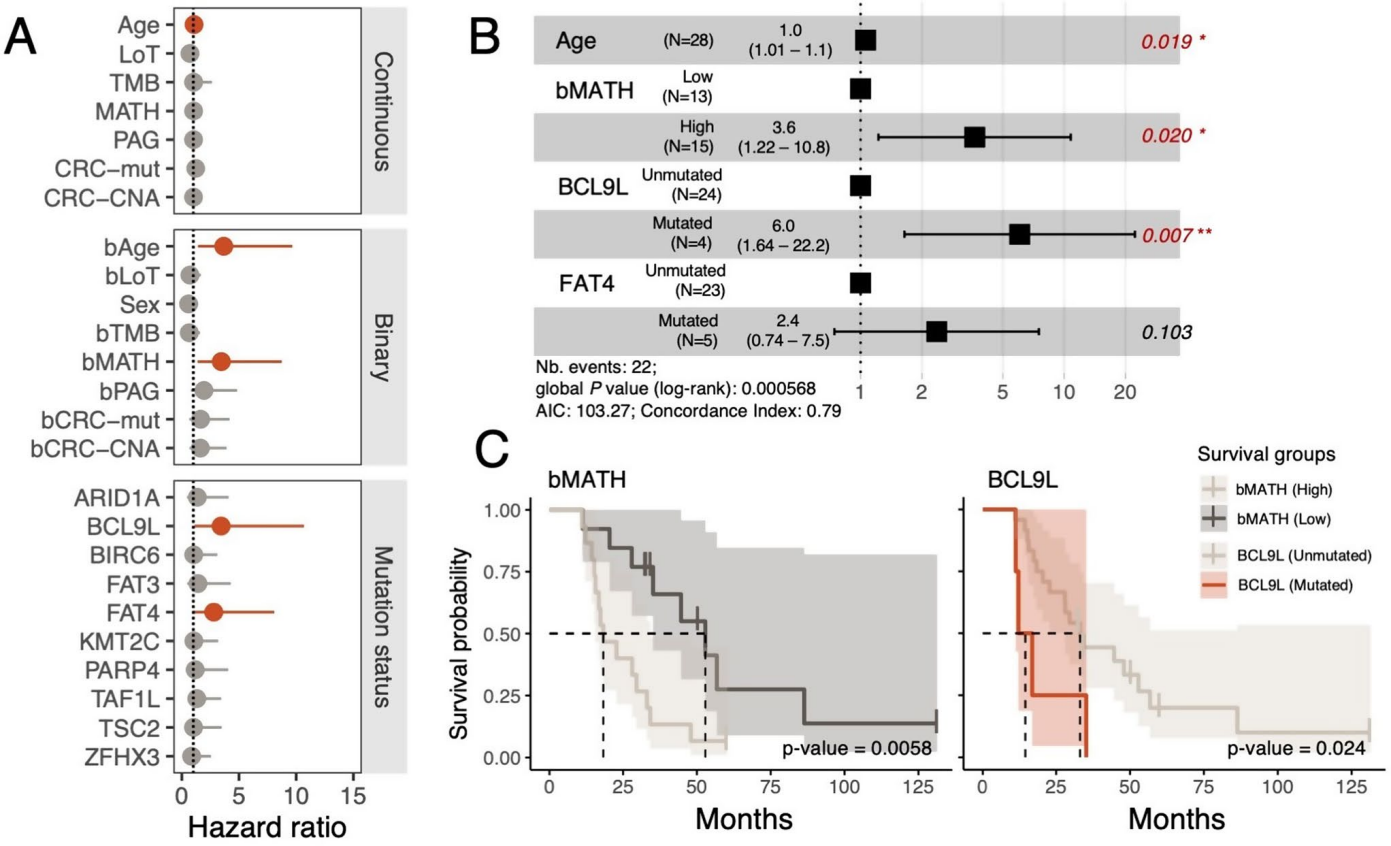
Impact of CTC genomic diversity on overall survival. **A**. Univariate CPH analysis for OS using all diversity metrics. Dots are the point estimate of the Hazard ratio, while the lines represent the 95% confidence interval (CI). The “Low” group was used as a reference in the binary version of all continuous variables. Hazard ratios with p-values < 0.1 are colored in red. **B**. Multivariate CPH analysis of survival time for the significant metrics and covariates in the univariate CPH analysis shown in A. The forest plot shows a 95% CI of HRs and the covariate p-values. Significant p-values are colored in red. **C**. Kaplan–Meier survival curves and *p-values* for bMATH and *BCL9L* on the mCRC cohort. The shaded area around each survival curve depicts the 95% CI

The univariate analyses indicated that patient age is significantly associated with shorter OS, both when analyzed as a continuous variable (Hazard Ratio [HR] = 1.07, 95% CI: 1.02–1.12, *p* = 0.00894) and when dichotomized (HR = 3.67, 95% CI: 1.39–9.67, *p* = 0.00859) (Fig. 3A). Patients in the High-bMATH group (HR = 3.45, 95% CI: 1.36–8.75, *p* = 0.00895) also had poorer OS compared to those in the corresponding “Low” group.

Notably, out of the 10 recurrently mutated genes evaluated, only *BCL9L* and *FAT4* were significantly associated with OS. Patients carrying non-silent mutations in *BCL9L* (*N* = 4) exhibited significantly worse OS than those without these mutations (HR = 3.71, 95% CI: 1.18–11.7, *p* = 0.02). Interestingly, these mutations in *BCL9L* were present at relatively high frequencies in the CTC pools, ranging from 0.180 to 0.727 (Fig. S1A), consistent with clonal or near-clonal events. Similarly, patients harboring non-silent mutations in *FAT4* (*N* = 5) also had a worse prognosis (HR = 2.80, 95% CI: 0.966–8.09, *p* = 0.058), with allele frequencies ranging from 0.104 to 0.176 (Fig. S1A). Importantly, patients with *BCL9L* or *FAT4* mutations did not differ significantly from the rest of the cohort in terms of other clinical or genomic features, including age, TMB, number of mutations in CRC driver genes, CNAs affecting CRC driver genes, or number of prior treatments (Fig. S1B). These comparisons suggest that the association of *BCL9L* and *FAT4* with OS is unlikely to be explained by other clinical or molecular factors.

To further assess the prognostic significance of these findings, we next performed a multivariate CPH analysis including patient age, bMATH, and the mutational status of *BCL9L* and *FAT4*, specifically, the variables that showed a significant association with OS in the univariate analysis (Fig. 3B). This model confirmed that both high bMATH and mutated *BLC9L* status were significant prognostic factors, with HRs of 3.6 (95% CI = 1.22–10.8, p-value = 0.020) and 6.0 (95% CI = 1.64–22.2, p-value = 0.007), respectively. Accordingly, the survival curves indicate that patients in the Low-bMATH group survive much longer (median survival time: 52.9 months) than their High-bMATH counterparts (median survival time: 18.3 months) (Fig. 3C). Similarly, patients carrying *BCL9L* mutations experienced a notable reduction in OS (median survival time: 14.5 months) compared to those without them (median survival time: 33.1 months).

### CTC diversity from gene panels can also predict patient survival

**Figure 4:**
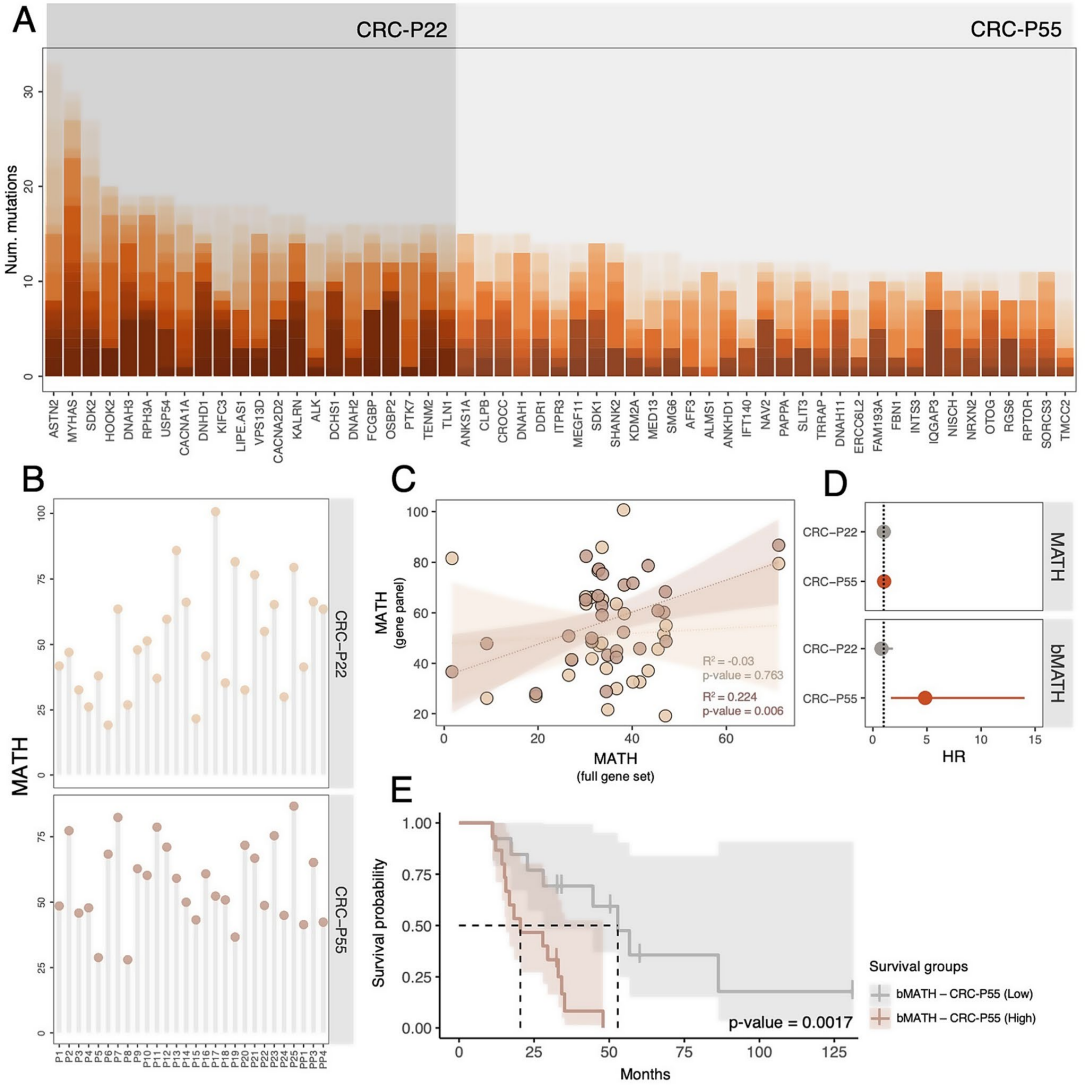
Optimized gene panel analysis. **A**. Genes included in the CRC-P22 and CRC-P55 panels were sorted according to mutation counts. Different color shades represent the different patients. **B**. MATH scores for each panel and patient. **C**. Scatter plot describing the similarity of MATH scores between gene panels and WES. Solid lines represent the best fit from regression analysis. R^2^ scores and *p-values* are shown on the bottom right side of the plot. Lighter and darker shades of brown highlight the MATH scores from CRC-P22 and CRC-P55, respectively. **D**. Univariate CPH analysis of survival time and MATH and bMATH for both gene panels. Dots are the Hazard ratio (HR) point estimate, while the lines represent the 95% CI. In the bMATH, the “Low” group was used as a reference. P-values < 0.05 are colored in red. **E**. Kaplan–Meier survival curves and *p-values* for bMATH of CRC-P55 gene panel. The shaded area around each survival curve depicts the 95% CI

To explore a practical and low-cost alternative for CTC genomic profiling, we designed two gene panels – CRC-P22 and CRC-P55 – each comprising the most frequently mutated genes in our cohort (Fig. 4A). The MATH scores derived from the CRC-P55 panel were highly correlated with those obtained from the WES data, whereas the CRC-P22 panel showed weaker concordance (Fig. 4B–C). Importantly, MATH estimates from the CRC-P55 panel were significantly associated with worse OS (Fig. 4D), both as a continuous variable (HR = 1.04, 95% CI: 1.01–1.07, *p* = 0.0223) and as a binary variable (HR = 4.83, 95% CI: 1.66–14.0, *p* = 0.00377). Patients in the high-bMATH group exhibited a significantly shorter median OS (20.4 months) compared to those in the low-bMATH group (52.9 months) (Fig. 4E). On the other hand, neither of the commercial panels showed a significant association between MATH scores and OS (Fig. S2).

## Discussion

The prognostic value of CTC enumeration has already been established, with higher CTC counts linked to aggressive disease across multiple cancer types, including CRC [20]. However, CTC populations can be genomically heterogeneous [24], a factor that, in the case of primary tumors, has already been linked to a poor prognosis [9–11].

Here, we found that, in mCRC, CTC populations with higher MATH scores - a measure of genomic diversity - or with non-silent mutations in *BCL9L* are significantly associated with poorer overall survival. To our knowledge, this is the first study showing an association between CTC genomic diversity and cancer outcome. Intuitively, larger overall genetic variation, as measured by the MATH scores, likely reflects the presence of distinct subclones, some of which could proliferate more rapidly, have a higher propensity for metastasis, or better evade cancer drugs [41]. In this context, genomic diversity estimates derived from CTC pools may not only serve as prognostic biomarkers but might also contribute to real-time monitoring of disease progression, treatment resistance, and metastatic potential (through longitudinal sampling). Increasing CTC heterogeneity over time could signal the emergence of resistant clones, potentially allowing for earlier clinical intervention. Conversely, a decline in diversity following treatment might suggest a favorable response. As demonstrated in this study, such information can be obtained non-invasively through a simple blood draw, providing a practical alternative to tissue biopsies, which are often difficult to obtain in metastatic settings.

Since the MATH scores in this study were derived from whole-exome sequencing, which may be impractical in clinical settings due to high costs and complex bioinformatics workflows [42], we explored the potential of smaller gene panels as an alternative. We found that MATH scores derived from a 55-gene panel (CRC-P55), based on the most frequently mutated genes in our cohort, were significantly associated with OS. In contrast, two commercial gene panels, Oncomine and oncoReveal, did not show any association. These findings highlight the potential of targeted panels to capture meaningful genomic diversity from liquid biopsy data. However, because CRC-P55 was constructed from a larger dataset already associated with OS, its prognostic performance may result from an ascertainment bias. As such, future validation in independent cohorts will be needed to confirm its broader clinical utility.

Importantly, we also observed that non-silent mutations in *BCL9L* were associated with reduced OS. *BCL9L* has previously been identified as a key mediator of aneuploidy tolerance in colorectal cancer, promoting genomic instability and tumor progression [43], a feature already recognized as a poor prognostic marker across several cancer types [44]. In our cohort, although patients with *BCL9L* mutations exhibited a higher fraction of CNAs, both the PAG score and CNA burden at CRC driver genes were not independently associated with poor survival.

Moreover, *BCL9L* is an essential co-activator in the Wnt/β-catenin signaling pathway [45]. Aberrant activation of this pathway is a common oncogenic event in CRC [46], driving tumor progression by facilitating epithelial-mesenchymal transition (EMT), which promotes invasion and metastatic potential. Interestingly, previous studies in murine models have shown that *BCL9L* knockout reduces stemness and tumorigenicity by inducing cellular differentiation [47]. In this context, if the non-silent *BCL9L* mutations in our cohort represent gain-of-function events, they could dysregulate β-catenin transcriptional activity, thereby promoting EMT and stem-like traits, and ultimately driving more aggressive phenotypes consistent with the poorer clinical outcomes observed in our cohort. Nonetheless, functional studies will be required to experimentally validate the phenotypic consequences of the *BCL9L* mutations.

An intriguing observation in our study was the rarity of somatic non-silent mutations in several key CRC driver genes, including *TP53*, *APC*, and *KRAS*. While *TP53* exhibited widespread CNAs across many CTC pools (Fig. 2C), suggesting potential gene disruption through alternative mechanisms other than point mutations, only a single *APC* and a single *KRAS* mutation were detected in the MSS patients. However, it is important to note that both *APC* and *KRAS* showed lower sequencing coverage compared to the average coverage across all genes in the CTC pools, with *KRAS* frequently falling below 20. This limited coverage likely reduced our sensitivity for detecting mutations in these genes (Fig. S3A). Furthermore, although multiple candidate non-silent mutations were initially identified in these genes, most were filtered out during post-calling processing (Fig. S3B). As a consequence, the reduced mutation burden observed in these genes likely reflects technical limitations.

Finally, it is important to acknowledge that our study has some limitations. First, the relatively small sample size (29 patients) may limit statistical power and reduce the generalizability of our results. This limitation is particularly relevant for MSI tumors, which were underrepresented in our cohort (*N* = 1), thus precluding any meaningful subgroup analysis. Second, we were unable to compare the prognostic impact of MATH and *BCL9L* mutational status with that of established clinicopathological markers, such as primary tumor sidedness [48], which was not collected for this cohort. In any case, future studies involving larger and well-annotated cohorts will be essential to validate our observations and assess their broader applicability in a clinical context.

In summary, our study demonstrates for the first time a relationship between CTC genomic diversity and overall survival in mCRC, underscoring the potential of genomic profiling of CTCs as a novel prognostic tool in mCRC. These findings highlight the need for further research into the clinical applications of CTC genomics, which could ultimately improve personalized treatment strategies for cancer patients.

## Electronic supplementary material

Below is the link to the electronic supplementary material.

Supplementary Material 1

Supplementary Material 2

## Data Availability

Raw exome sequencing data from CTC pools, together with matching healthy samples, have been deposited in the Sequence Read Archive database under the accession code PRJNA1278982. All data supporting the findings of this study are available within the article and its supplementary information files.
