# Supplementary material for: Genomic diversity and BCL9L mutational status in circulating tumor cells predict overall survival in metastatic colorectal cancer
Source: Cell Oncol (Dordr). 2025 Oct 6;48(6):1809–20. doi: 10.1007/s13402-025-01109-x (PMC12698839; doi:10.1007/s13402-025-01109-x)
Supplement: Supplementary file 1 — Supplementary Material 1 [file 13402_2025_1109_MOESM1_ESM.pdf]

Supplementary information

Genomic diversity and *BCL9L* mutational status in circulating tumor cells predict overall survival in metastatic colorectal cancer

Joao M. Alves[1,2,#,], Nuria Estévez-Gómez[1,2,#], Roberto Piñeiro[3,4], Laura Muinelo-Romay[4,5], Patricia Mondelo-Macía[5], Mercedes Salgado[6], Agueda Iglesias-Gómez[7], Laura Codesido-Prada[7], Astrid Diez-Martín[7], Joaquin Cubiella[7], David Posada[1,2*]*

[1]CINBIO, Universidade de Vigo, 36310 Vigo, Spain.
[2]Galicia Sur Health Research Institute (IIS Galicia Sur), SERGAS-UVIGO, Vigo, Spain.
[3]Translational Medical Oncology Group, Oncomet, Health Research Institute of Santiago de Compostela (IDIS), Santiago de Compostela, Spain
[4]Centro de Investigación Biomédica en Red de Cáncer (CIBERONC), Madrid, Spain.
[5]Liquid Biopsy Analysis Unit, Translational Medical Oncology Group, Health Research Institute of Santiago de Santiago de Compostela (IDIS), Santiago de Compostela, Spain
[6]Department of Oncology, Hospital Universitario de Ourense, Research Group in Gastrointestinal Oncology-Ourense, Ourense, Spain
[7]Department of Gastroenterology Hospital Universitario de Ourense, Research Group in Gastrointestinal Oncology-Ourense, Centro de Investigación Biomédica en Red de Enfermedades Hepáticas y Digestivas (CIBERehd), Ourense, Spain

[#] these authors contributed equally to this work

***Corresponding authors:** Joao M. Alves (jalves@uvigo.gal), David Posada (dposada@uvigo.es).

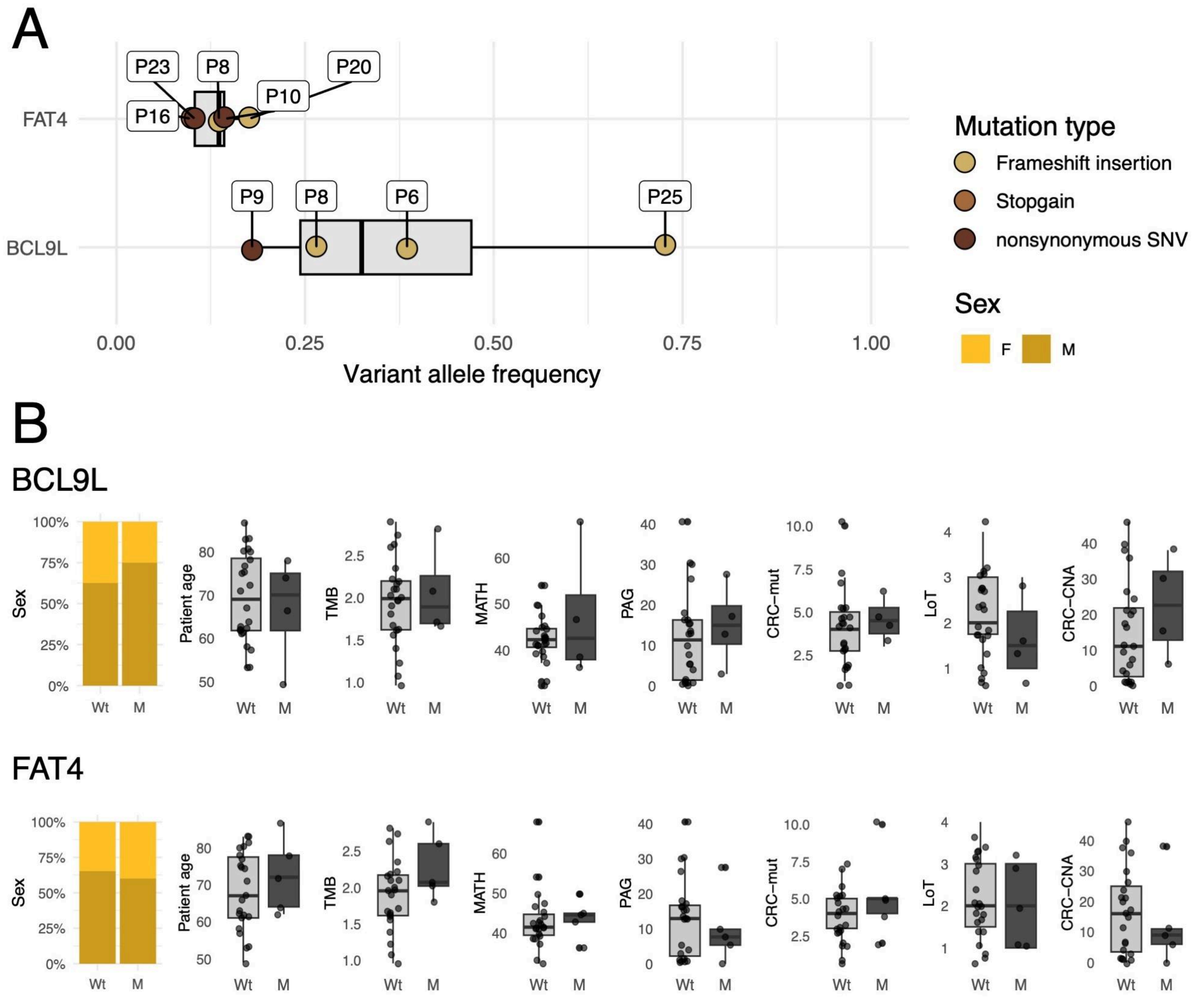

Figure S1. Allele frequency estimates and features of *BCL9L* and *FAT4* mutated patients. A. Allele frequency estimates of *BCL9L* (N=4) and *FAT4* (N=5) mutations. Individual mutations colored according to mutation type. Patient ID shown above each data point. **B.** Comparison of clinical and molecular features for patients with unmutated (Wt) and mutated (M) versions of *BCL9L* and *FAT4* genes. The bar plot on the left shows the proportion of female (light gold) and male (dark gold) patients in the Wt and mutated M groups. Boxplots display the distributions of additional clinical and molecular metrics between Wt and mutated M groups.

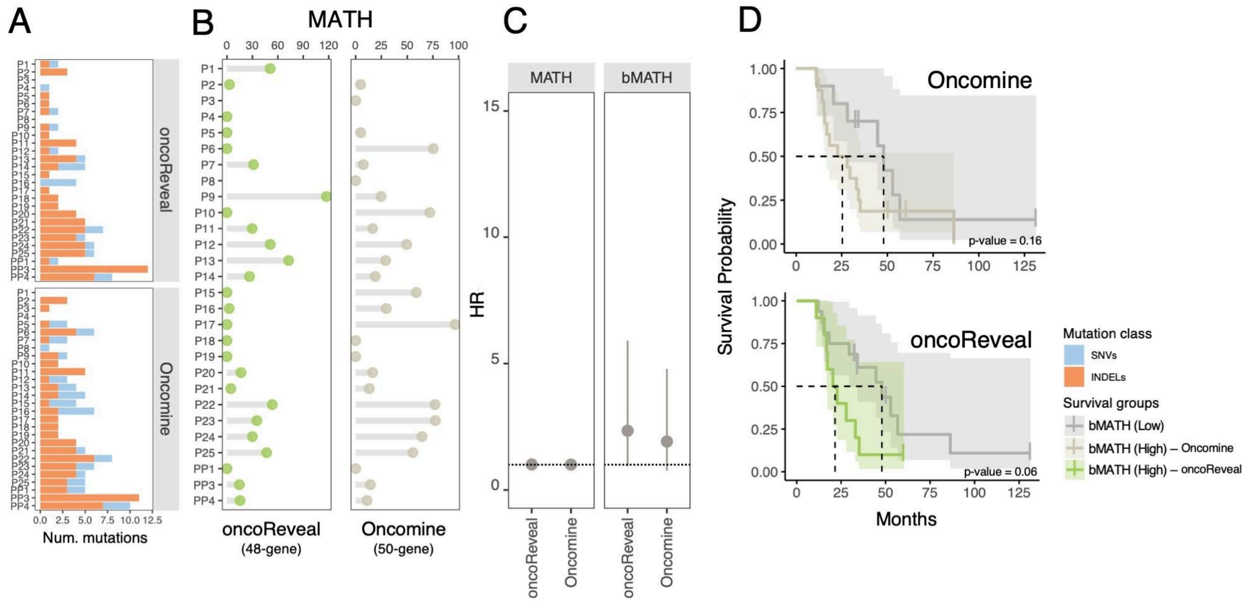

Figure S2. Commercial NGS panel analysis. A. Mutation counts in each gene panel, including SNVs (light blue) and indels (orange). **B.** MATH scores for each panel and patient. **C.** Univariate CPH analysis of survival time and MATH and bMATH for both gene panels. Dots are the point estimate of the Hazard ratio (HR), while the lines represent the 95% CI. In the bMATH, the "Low" group was used as a reference. **D.** Kaplan–Meier survival curves and p-values for the bMATH score of both gene panels. The shaded area around each survival curve depicts the 95% CI.

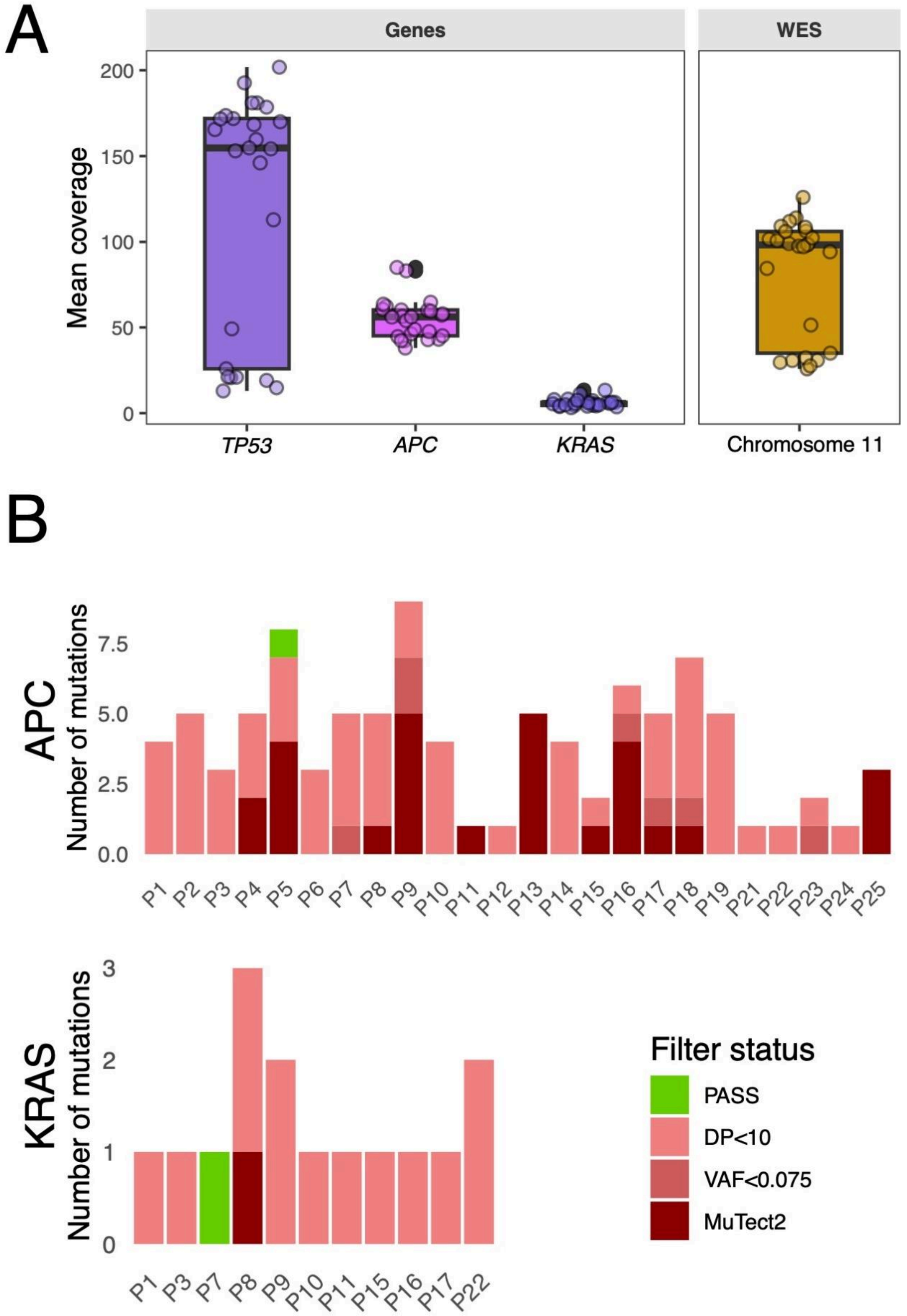

Figure S3. Coverage and mutational filtering at key CRC driver genes. A. Boxplots depicting the distribution of the mean sequencing coverage for TP53, APC, and KRAS genes across all CTC pools from microsatellite stable (MSS) patients. Each dot represents a single sample. For comparison, the right panel shows the mean coverage of chromosome 11 (which is mostly diploid in the cohort) used here as a representative reference for whole-exome coverage (WES). **B.** Stacked barplots showing the number of candidate non-silent mutations per patient in APC (top) and KRAS (bottom). Mutations are grouped by filtering status: PASS (green), low coverage (DP<10, light red), low allele frequency (VAF<0.075, medium red), or filtered by Mutect2 (dark red).
